# Doping-induced performance optimization in monolayer WS_2_ memristor: reduced variability and contact resistance†

**DOI:** 10.1039/d5ra02473k

**Published:** 2025-07-22

**Authors:** Tanshia Tahreen Tanisha, Orchi Hassan, Md. Kawsar Alam

**Affiliations:** a Department of Electrical and Electronic Engineering, Bangladesh University of Engineering and Technology Dhaka 1205 Bangladesh orchi@eee.buet.ac.bd kawsaralam@eee.buet.ac.bd

## Abstract

The memristor is a cornerstone for developing novel non-volatile memory devices that enable brain-like efficient processing and storage capabilities. Two-dimensional transition metal dichalcogenide (TMDC)-based memristors are gaining increasing attention due to the advantages they present over their bulk counterparts. In this work, we employed first-principles calculations to demonstrate that dopants play a significant role in reducing the cycle-to-cycle variability and in lowering the contact resistance in monolayer WS_2_-based memristor. The possibility of reduced cycle-to-cycle variability is reflected by the attractive nature of the calculated interaction energy between dopant metal atoms and a sulphur monovacancy in the WS_2_ monolayer. The potential for reduced contact resistance is evident from the reduced tunneling barrier heights and increased tunneling probabilities at the electrode/WS_2_ interface upon doping. Additionally, extra states are found to appear in the density of states upon doping, which can prove useful for adjusting the conductance of a doped WS_2_-based memristor as required. Finally, the obtained features are used to outline dopant selection criteria based on the valence electron configuration of dopants. The obtained characteristics and outlined criteria can serve as guidelines for the future design of optimized WS_2_-based memristive devices, possessing lower contact resistance and reduced variation in device performance.

## Abstract 2

Metal doping in monolayer WS_2_ induces strong dopant–vacancy interactions and improves tunneling at the Au electrode–WS_2_ interface, highlighting its potential to reduce variability and contact resistance in WS_2_-based memristors.

## Graphical abstract

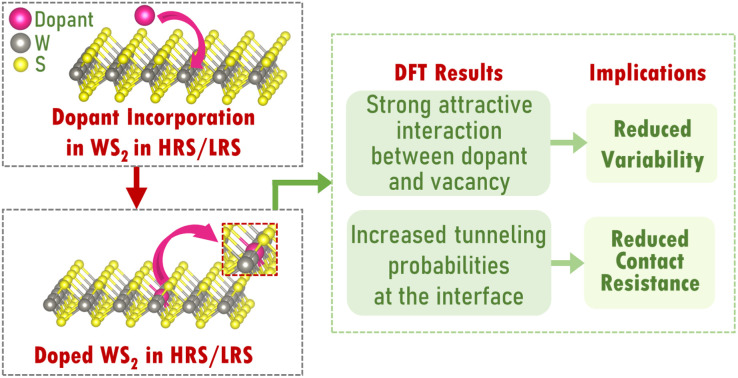

## 1. Introduction

The memristor was theoretically introduced as the fourth basic circuit element by Leon O. Chua^1^ in 1971, and it was shown that this element establishes the previously undefined relationship between the two fundamental circuit variables, charge and flux. The dependence of a memristor's resistance *i.e.* its memristance on past stimuli can be exploited to construct memory devices utilizing materials that possess memristive properties.^1,2^ Memristors have found applications in several areas, such as neuromorphic systems,^3–5^ in-memory computing,^6–8^ stochastic computing,^9,10^ logic circuits,^11–13^ radio frequency circuits,^14–16^*etc.* Moreover, the information stored in a memristor persists even when power is turned off, which enables the usage of memristors in non-volatile memory devices. In this context, resistive random-access memory (RRAM or ReRAM) stands as a prime candidate for advanced non-volatile memory devices, where the high and low resistance states (HRS and LRS) of a resistive switching layer correspond to logic 0 and logic 1, respectively^17–19^ or *vice versa*.^20,21^ Due to the non-volatile memory capabilities of memristors, they naturally lend themselves to RRAM applications.

Traditional RRAM devices that use oxides as the insulator material suffer from high voltage requirements and high switching times due to the 3D bulk nature of metal oxides. Two-dimensional materials have now emerged as compelling alternatives to metal oxides for use as the active materials in RRAM devices. The advantages that 2D materials offer over their 3D counterparts encompass scaled-down device dimensions,^22,23^ low power consumption,^24,25^ reduced threshold voltage,^26^*etc.* Owing to these merits, research is being actively pursued on several two-dimensional materials, such as BN,^27,28^ graphene,^29^ MoS_2_, and other transition metal dichalcogenides (TMDCs),^30^ heterostructures,^31^ and perovskites^32^ for their possible application in non-volatile RRAM devices. Moreover, memristors based on 2D materials have found application in artificial electronic synapses,^26,33–35^ artificial neurons,^36^ RF switches,^37^ logic gates for logic-in-memory calculations,^38^ neuromorphic computing,^39–41^*etc.*

In this work, we have used first-principles calculations to explore the potential of metal-doped monolayer WS_2_ for application as memristors, particularly as RRAMs, when arranged in a vertical conformation with Au electrodes. Our choice of WS_2_ stems from the outstanding properties of WS_2_, which are, a tunable bandgap of ∼2.1 eV,^42^ a very high electron mobility of 1103 cm^2^ V^−1^ s^−1^ (ref. 43) (important for low-power consumption), a high ON/OFF current ratio of ∼10^5^,^44^ and the prevalence of use of WS_2_ in several RRAM devices.^30,45–47^ Lateral architectures are widely used in experimental studies due to their fabrication simplicity and reduced risk of shorting *via* native defects such as sulphur vacancies. However, vertical structures with metal-monolayer–metal stacking have also been actively studied in recent years.^22,30,47^ Such structures have greater integration potential, smaller inter-electrode spacing, and lower switching voltage requirements, as demonstrated in several recent experimental works. While lateral devices often require larger switching voltages (20–100 V) due to micrometer-scale gaps between electrodes, vertical architectures using monolayer MoS_2_ and h-BN have achieved switching at low voltages (0.5–3 V) and enable high-density memory design.^30^ Owing to these advantages, in this study, we have particularly focused on WS_2_ memristor possessing a vertical metal-monolayer–metal stacking.

We were motivated to investigate the effect of doping on WS_2_-based memristor or RRAM in this work inspired by the different positive outcomes of doping observed for doped oxide-based memristors experimentally and theoretically. For instance, p-type dopants (Al, Hf, Zr, and Ti) were found to prove conducive to lowering the forming/set voltage and improving the retention properties of Ta_2_O_5_ ReRAM.^48^ In the case of TiO_2_-based resistive memory, dopants were found potentially useful for the reduction of forming voltage, reduction of RESET current, and realization of controlled and predictable percolation.^49^ Interactive energy calculations for HfO_2_-based RRAM revealed that the dopant metals have an attractive effect on the oxygen vacancies, and p-type substitutional metal dopants were found to have a strong enhanced effect on the oxygen vacancy filament.^50^ Al or La doping in ZrO_2_ led to the reduction in calculated oxygen vacancy formation energy, better control in vacancy formation, and improved uniformity in experimentally studied ZrO_2_-based RRAM devices.^51^

However, the effect of doping on 2D material-based memristor or RRAM has not been theoretically or experimentally explored as of yet, which motivated us to address the area in the present study. The performed interaction energy calculations in this study reveal that the dopants play an important role in reducing the cycle-to-cycle variability in WS_2_-based memristors. Such variability resulting from the stochastic nature of the switching process is a significant challenge in non-volatile resistive switching devices,^52,53^ and our results reveal that dopants can help address this concern. In addition, our computations unveil that dopants help reduce the contact resistance of WS_2_-based memristor by decreasing the tunneling barrier across the electrode/WS_2_ interface. To the best of our knowledge, this is the first theoretical study that addresses the concerns of cycle-to-cycle variability and contact resistance in the context of a 2D material-based memristor.

To identify how dopants influence the behavior of WS_2_-based memristor, we have worked with a total of 11 metal dopants, which are Sr, Al, Ga, In, Ti, Zr, Hf, Nb, Mo, Re, and Ru, encompassing both n-type and p-type dopants. In the discussion that follows, at first, we constructed the structures corresponding to the HRS and LRS of a WS_2_ memristor. Following this, calculation of interaction energies, tunneling probabilities, tunneling barrier heights, and density of states are performed for the doped systems to unravel the changes in attributes that doping brings into effect in monolayer WS_2_-based memristor. Finally, some dopant selection criteria are proposed based on the obtained results, which are expected to enable the design of optimized WS_2_-based memristors in the future.

## 2. Results and discussions

This section consists of seven sub-sections, numbered 2.1 to 2.7. In Section 2.1, we present the structures used for representing a WS_2_-based memristor (in HRS and LRS), which are used for subsequent calculations. Dopants are brought into the picture in Section 2.2, and the structures used for representing the high and low resistance states of doped WS_2_ are presented here. Following this, in Section 2.3, the possibility of reduced cycle-to-cycle variability upon doping is presented from interaction energy calculations. Next, in Section 2.4, it is demonstrated that dopants help lower the contact resistances at the interface by lowering the tunneling barrier heights seen by electrons and increasing the corresponding tunneling probabilities. The density of states for the doped configurations are analyzed in Section 2.5. In Section 2.6, the effect of lowering doping concentrations on the key characteristics is laid out for dopants that have a high mismatch of ionic radius with the host atom, W. In the end, dopant selection criteria are outlined in Section 2.7 based on the inferences obtained in Sections 2.1 to 2.6. The two main outcomes of this section are the implications found for reduced cycle-to-cycle variability (Section 2.3) and lower contact resistances (Section 2.4) in WS_2_ memristor upon doping.

### 2.1. Structural models for the undoped high and low resistance states

**Fig. 1:**
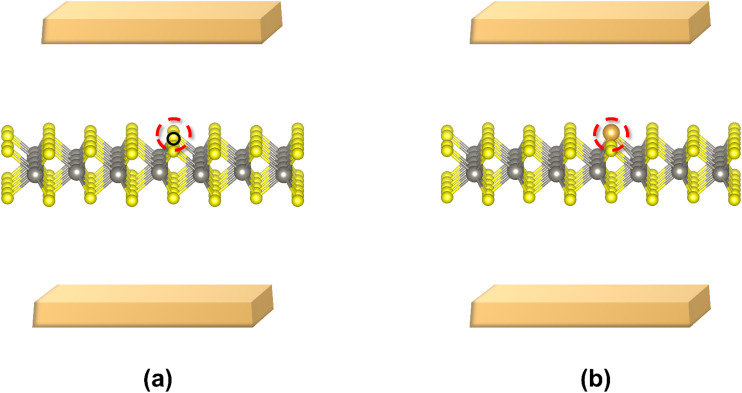
Schematic of WS_2_-based memristor (a) in the HRS, without an Au conductive-point, (b) in the LRS, with a conductive-point. The small black circle in (a) represents the vacancy in the HRS, the red dashed circle encircling it is added to further highlight the vacancy. The red circle in (b) highlights the Au conductive point. The top and bottom golden blocks represent Au electrodes.

We constructed the structural models of undoped high and low resistance states of monolayer WS_2_ memristor based on the conductive-point mechanism, which we validated as the underlying resistive switching mechanism in monolayer WS_2_-based memristors based on calculations detailed in Section B of the ESI.† A 4 × 4 × 1 supercell of WS_2_ containing a sulphur monovacancy has been constructed as the structure corresponding to the high resistance state. Conversely, a 4 × 4 × 1 supercell of WS_2_ with a single Au atom adsorbed at the sulphur monovacancy site has been constructed as the structure for the low resistance state, where the Au atom represents the conductive-point. The schematics of the high resistance and low resistance states (HRS and LRS) corresponding to the conductive-point mechanism are shown in Fig. 1. These structures are used for the calculations that follow.

Several vacancies occur in monolayer WS_2,_ such as W vacancy (V_W_), S monovacancy (V_S_), S divacancy (V_S_2__), and WS_3_ vacancy (V_WS_3__). Among them, V_S_ was considered for the HRS and LRS structures here due to its greatest probability of existence and hence the greatest vacancy concentration, as suggested by our formation energy calculations. Moreover, sulphur vacancies were experimentally found to be the most abundant ones in WS_2_.^54^ The details of formation energy calculations are laid out in Section A of the ESI.†

### 2.2. Dopant-incorporated configurations for the HRS and LRS

**Fig. 2:**
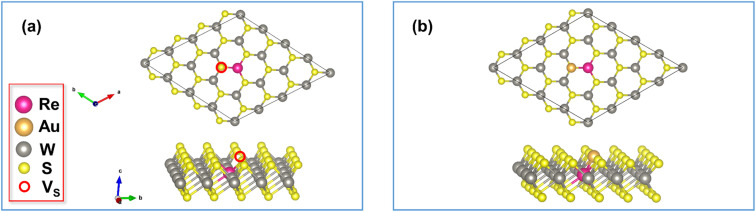
Structures of (a) Re-doped WS_2_ in the HRS and (b) Re-doped WS_2_ in the LRS.

In this work, we have considered the following metal dopants: Sr, Al, Ga, In, Ti, Zr, Hf, Nb, Mo, Re, and Ru. Among these, WS_2_ monolayers doped with Al, Ga, In, Nb, Mo, and Re have already been experimentally synthesized.^55–59^ Apart from these, Ru was experimentally used as a dopant with WS_2_ nanosheets.^60^ Moreover, Ti, Zr, and Hf-doped WS_2_ have been investigated in earlier theoretical works,^61^ albeit in a different context. In Section C of the ESI,† we assessed the compatibility of Sr, Ti, Zr, and Hf with WS_2_ in terms of ionic radius, since these four metals were not experimentally studied as dopants for monolayer WS_2_ in earlier works, and found that they are all potentially compatible with WS_2_ for substitutional doping. To obtain the doped structures, we substituted a single W atom in the 4 × 4 × 1 supercell with a metal dopant atom, resulting in a dopant concentration of 6.25% or ∼6.15 × 10^13^ cm^−2^ (the exact value varies slightly with the type of dopant since the doped structures have different values of the optimized cell parameters). This is consistent with the comparable doping concentrations chosen for theoretical and experimental studies on doped WS_2_.^56–59,61–63^ The structures corresponding to the HRS and LRS of metal-doped WS_2_ formed in this way are shown in Fig. 2(a) and (b), respectively with Re as the representative metal-dopant.

In the doped structures, the metal dopant atom was placed at the minimum energy site, and the relevant calculations are elaborated in Section D of the ESI.†

### 2.3. Reduced cycle-to-cycle variability stemming from localization of vacancies due to attractive dopant–vacancy interaction energy

**Fig. 3:**
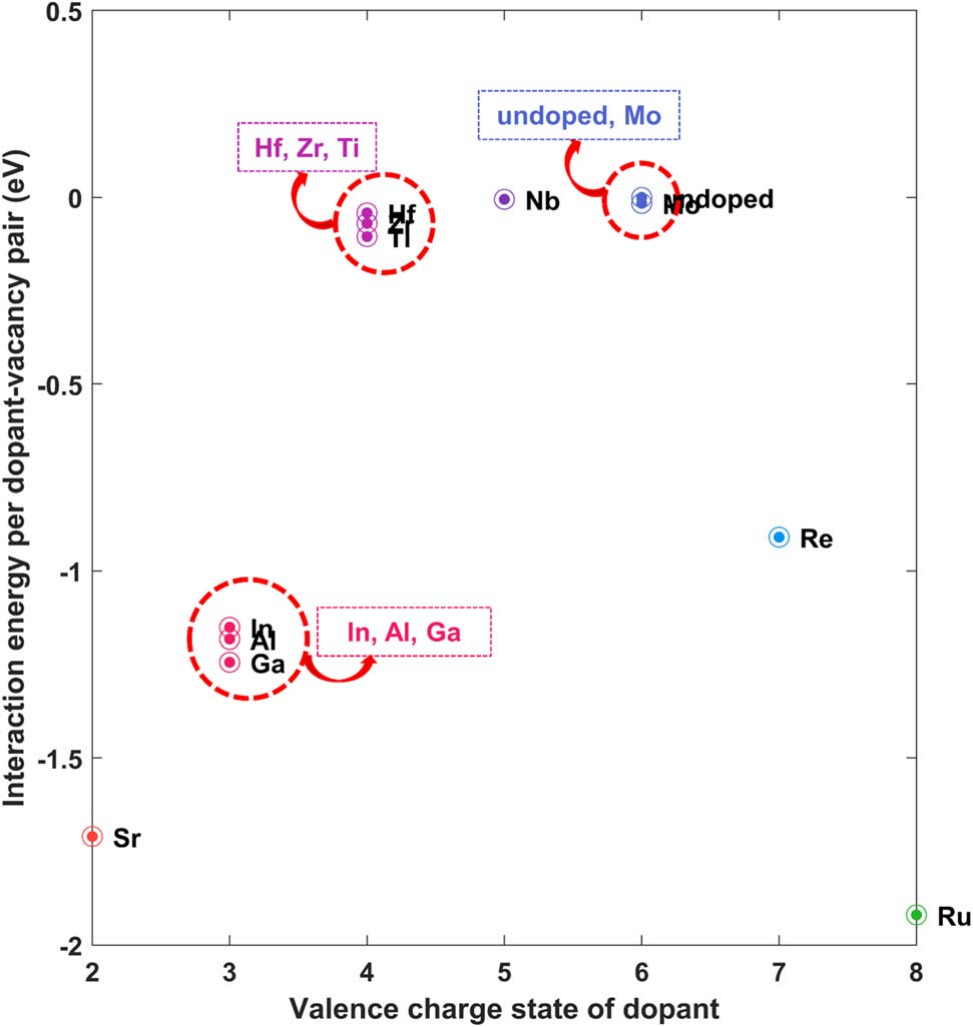
Interaction energy per dopant–vacancy pair (with SOC) between dopant metal atom and sulphur monovacancy in doped WS_2_.

According to the valence electron number, dopants can be classified into three kinds: p-type, n-type, and W-like. This categorization is laid out in Table S5 of the ESI.† At first, we calculated the interaction energies between dopant atoms and sulphur monovacancies to extract information regarding the influence of dopant incorporation in WS_2_ memristors. We considered only the HRS structures for these calculations since we are interested in the interaction between the vacancy and the dopant atom, for which the HRS structure suffices. The calculated values are laid out in Table S5,† taking spin–orbit coupling (SOC) into account. For illustration, the values are depicted in Fig. 3. From Fig. 3, it is easily noticed that the interaction energy assumes negative values for all dopants. Based on our definition of interaction energy, a positive interaction energy signifies a repulsive interaction between V_S_ and the corresponding dopant metal. The negative values of interaction energies observed for all dopants signify an attractive interaction between the dopant and the vacancy. Dopants that have a greater difference in the number of valence electrons (Sr, Al, Ga, In, Ru) with W *i.e.* stronger p-type and n-type dopants correspond to higher magnitudes of the interaction energies, implying a stronger attraction with V_S_. On the other hand, the dopants with a number of valence electrons close to 6 (number of valence electrons of W) *i.e.* Hf, Zr, Ti, Mo, Nb, Re are related to relatively lower values of dopant–vacancy interaction energy. These weaker dopants thus result in an attraction that is not as strong as in the case of strong dopants. For application in RRAM, this strong attraction could be useful because the dopants can attract the vacancies and keep them localized. The sulphur (or other chalcogen) vacancies in TMDCs are mobile in nature^64,65^ and keeping them bound to a location through strong attraction can help keep the vacancy position unchanged from one cycle of device operation to another, and thus play an instrumental role in reducing cycle-to-cycle variability. Thus, dopants could play a pivotal role in reducing randomness in conductive-point formation and reducing fluctuations in device parameters. Such notable influence of dopants was also observed in TiO_2_-based resistive memory.^49^

The conclusions drawn from interaction energy calculations highlight the pivotal role played by dopants in addressing the major concern of cycle-to-cycle variability in non-volatile resistive switching devices.

### 2.4. Implications for lowering of contact resistances at the Au/WS_2_ interface upon doping

**Fig. 4:**
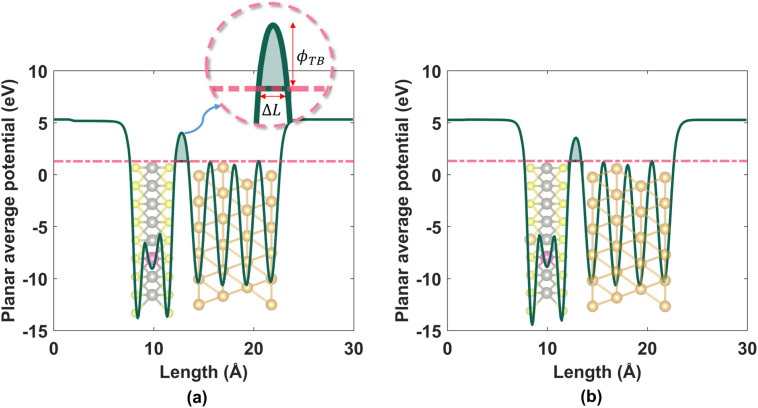
Planar average potentials for (a) Re-doped WS_2_ in the HRS and (b) Re-doped WS_2_ in the LRS.

Next, we calculated the tunneling barrier heights, widths, and tunneling probabilities related to the tunneling potential barrier seen by electrons at the Au/WS_2_ interface for each of the 11 dopants in the HRS and the LRS. We conducted this analysis to understand the effect of different dopants on the interfacial properties (tunneling barrier height, tunneling probability, and contact resistance) of WS_2_-based memristor. The HRS and LRS structures that were used for these calculations are shown in Fig. S3(a) and (b)† respectively for the representative case of Re dopant. The obtained profiles of planar average potential are shown for the representative case of Re dopant in Fig. 4.

These profiles were then used to calculate the tunneling barrier heights, widths, and tunneling probabilities in the HRS and LRS, and the values (with SOC taken into account) are laid out in Table 1.

**Table 1:** Tunneling probabilities (with SOC) in the HRS and LRS of metal-doped WS_2_

Dopant	*T* in HRS (T_HRS_) (%)	*T* in LRS (T_LRS_) (%)
Sr	30.49	35.75
Al	27.05	33.97
Ga	28.21	34.08
In	29.19	30.01
Ti	26.17	34.27
Zr	27.10	34.58
Hf	26.36	33.73
Nb	25.86	32.10
W (pure 4 × 4)	25.28	25.89
Mo	25.81	31.15
Re	25.74	30.35
Ru	28.56	29.22

Table 1 demonstrates that *T* is higher in the HRS for all 11 dopants compared to the corresponding undoped case. Moreover, the higher up or lower down the dopant element is positioned in Table 1, the greater the increase in *T* from its value in the undoped scenario. Higher values of *T* correlate to lower contact resistances, facilitating the development of more efficient, low-power devices. In the case of LRS, the same phenomenon is observed.

To aid visualization, the values of *T*_HRS_ and *T*_LRS_ for the 11 different dopants are plotted against the valence charge state of the dopants in Fig. S4 of the ESI.† From Fig. S4(a),† it is evident that *T*_HRS_ for each dopant is higher than that of the undoped case, consistent with the data in Table 1. It can also be seen that, as we move further away from W in the number of valence electrons, *T*_HRS_ generally keeps increasing. Fig. S4(b)† reveals that *T*_LRS_ is also higher than the undoped case for all of the dopants. As we go from the valence charge state of 2 to 8, *T*_LRS_ generally reduces, although In deviates from this trend. We are emphasizing higher values of *T* since they indicate the possibility of reduced contact resistance, and hence better device performance. The results presented in this section underline the significant role played by dopants in lowering the contact resistance of WS_2_-based memristors and thereby enhancing device performance.

### 2.5. Impact of different dopants on the density of states

**Fig. 5:**
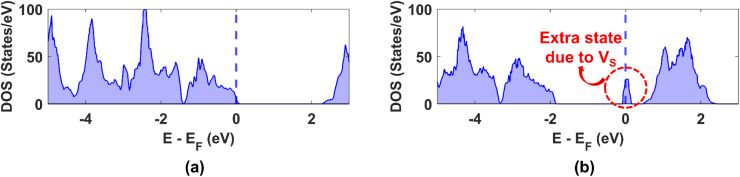
Density of states plot for 4 × 4 × 1 supercell of undoped WS_2_ (a) devoid of any vacancy and (b) containing a sulphur vacancy, V_S_.

The obtained DOS for undoped WS_2_ without any vacancy and with a single sulphur vacancy, V_S_ are calculated with HSE06 functional and depicted in Fig. 5. An extra state appears in the bandgap in Fig. 5(b), which is not visible in Fig. 5(a). Thus, this extra state can be attributed to the V_S_ vacancy. This information will prove useful for later discussions.

Next, we investigated the density of states (DOS) for both the high and low resistance states in the presence of each of the 11 dopants considered in this work using the structures shown earlier in Fig. 2(a) and (b). In this case, to isolate the effect of SOC, we computed the DOS with PBE functional both without and with SOC. The DOS corresponding to the HRS and LRS of the doped structures are presented in Tables S6 and S7 of the ESI,† respectively.

Let us look at the DOS without SOC for HRS in the absence of dopants (first row of Table S6†). A defect state (encircled) appears to exist in the bandgap right at the Fermi level, which can be attributed to the single sulphur vacancy as explained earlier from Fig. 5. The same inference was drawn in ref. 45 When SOC is taken into account, a similar DOS is obtained, with the only difference that the distance between conduction and valence band reduces when SOC is considered, as labelled in Table S6(a).† A close inspection reveals a similar observation for each dopant, except for Re. In the case of Re, two extra states appear in the bandgap of the DOS without SOC and three states in the DOS with SOC, but the shape remains the same in both cases. Thus, we only explain the DOS with SOC hereon for each dopant.

For the isovalent dopant Mo (Table S6(b)†), the DOS is similar to the undoped scenario, except for the fact that the Fermi level shifts closer to the valence band. The similarity in DOS indicates that undoped WS_2_ and Mo-doped WS_2_ have similar electronic properties, which explains the similar properties obtained for the two kinds of structures so far.

On the other hand, the appearance of two additional energy states corresponding to the metal dopant atom and bandgap reduction are visible for the n-type dopants Re (Table S6(c)†) and Ru (Table S6(d)†), explaining why *T* keeps increasing in the HRS as we move further down Table 1 from W to Mo, Re, and Ru.

In the case of the p-type dopant Nb, the Fermi level moves into the valence band (Table S6(e)†). Two distinct defect states appear in the bandgap, and they can be attributed to the vacancy and to the metal dopant itself. For Ti, Zr, and Hf, 2–3 distinct levels can be identified within the bandgap (Table S6(f)–(h)†), which also result in a reduced overall bandgap. In the case of In (Table S6(i)†), the Fermi energy moves a little away from the valence band and into the conduction band, which is indicative of metallization. A total of five defect states appear in the DOS arising from the vacancy and the dopant atoms. Three lie between the Fermi level and conduction band, one lies between the Fermi level and the valence band, and the other lies right along the Fermi energy. For Ga and Al (Table S6(j) and (k)†), a similar phenomenon is observed, with the exception that the number of defect states above the Fermi energy becomes two. Inter-gap states arising from Sr-doping are four in number, and three of them lie above the Fermi energy (Table S6(l)†). The other one lies along the Fermi energy. Overall, as we go higher or lower down the list of dopants in Table 1 starting from undoped WS_2_, the number of dopant energy states increases in general and the bandgap reduces, which explains the increased tunneling probability, *T* in the HRS for all 11 dopants. The defect states arising in the DOS due to dopants are encircled in red in Table S6(a)–(l).†

Now, we take a look at the DOS for LRS in the absence of dopants (Table S7†).

In this case too, the DOS without and with SOC are similar, with the only difference of reduced gap between conduction and valence bands upon taking SOC into account. As observed in Table S7(a)† (with SOC), the bandgap appears lower in the LRS compared to the HRS shown in Table S6(a),† which explains the increased conductivity in the LRS. In addition to the vacancy-included defect state at the Fermi level, another defect state, merged almost with the deep levels of the conduction band appears in the LRS, which is absent in the HRS, indicating that the Au atom in the LRS contributes to this state, further enhancing the conductivity in the LRS. For all dopants, the difference in the DOS of HRS and the corresponding LRS is not too different, and the difference in conductivity between the two states cannot be explained from DOS alone. The additional states arising in the DOS of LRS due to the dopants are encircled in green in Table S7(a)–(l).†

Since including SOC in HSE06 calculations of supercells is highly computationally expensive, we next performed the HSE06-based DOS calculations without SOC to isolate the impact of the hybrid functional, which are presented in Fig. S5 and S6 of the ESI† for the HRS and LRS, respectively. The DOS in Fig. S5 and S6† reveal that they are similar in shape to those shown in Tables S6 and S7,† respectively. However, the bandgap increases significantly for HSE06-computed DOS compared to GGA-computed ones. Thus, the GGA *vs.* GGA + SOC comparison highlights that the effect of SOC is to reduce the bandgap compared to the case without SOC, while the HSE06 (without SOC) results reflect that a more accurate exchange–correlation treatment leads to an increase in the bandgap with no additional significant changes.

In addition to explaining why *T* increases in the HRS and LRS upon doping, the computed DOS reveal that the strong n-type and p-type dopants lead to too much reduction of the bandgap even in the HRS, which could lead to leakage currents and thus should not be resorted to in a practical scenario. The DOS plots help further infer that the conductivity in the HRS and LRS can be continuously tuned through the use of different kinds of dopants, since different dopants introduce different inter-gap states and thus modulate the conductivity of the materials.

### 2.6. Effect of lower doping concentrations on the obtained characteristics

Given the significant differences in ionic radii between several dopants and W, achieving a doping concentration of 6.25% in practical experiments may be challenging. In this section, we verify whether the conclusions outlined thus far remain valid when the supercell size is expanded to reduce the doping concentration. We chose Sr, Al, Ga, In, Ti, Zr, and Re as the representative dopants since they are among the dopants with maximum ionic radii mismatch with W.

Firstly, we computed the interaction energies (including SOC) for a lower doping concentration to verify whether our conclusion regarding cycle-to-cycle variability remains valid. To do so, we chose a 5 × 5 × 1 supercell of WS_2_ with one dopant atom instead of the previous 4 × 4 × 1 one, thus leading to 4% doping concentration, which is lower than the 6.25% chosen before. The obtained interaction energies are laid out in Table 2. The energies calculated earlier for the 4 × 4 × 1 are also tabulated to enable easy comparison.

**Table 2:** Values of dopant–vacancy interaction energy in 4 × 4 × 1 and 5 × 5 × 1 doped and undoped supercells of WS_2_ and their difference

Dopant	*E*_int_ (4 × 4 × 1) (eV)	*E*_int_ (5 × 5 × 1) (eV)	Difference (eV)
Sr	−1.7104	−1.7126	0.0022
Al	−1.1822	−1.1644	0.0178
Ga	−1.2435	−1.2107	0.0328
In	−1.1510	−1.1528	0.0018
Ti	−0.1051	−0.1193	0.0142
Zr	−0.0695	−0.0863	0.0168
W (undoped)	0.0000	0.0000	0.0000
Re	−0.9094	−0.9322	0.0228

**Fig. 6:**
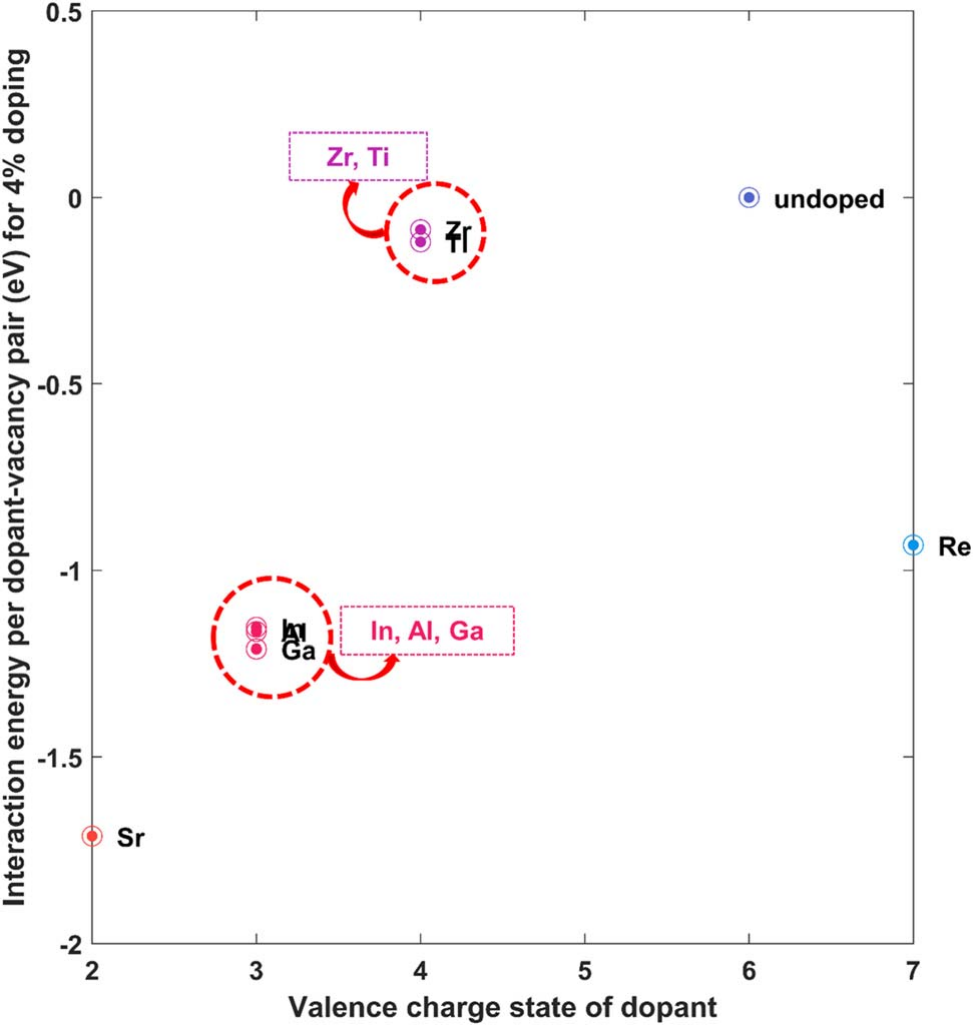
Interaction energy per dopant–vacancy pair (with SOC) between dopant metal atom and sulphur monovacancy in WS_2_ at lower doping concentration of 4%.

From Table 2, it can be observed that the interaction energies obtained for the 4 × 4 × 1 and 5 × 5 × 1 supercells are quite close to each other. In each case, the difference in energies is minimal (fourth column of Table 2). For Sr, Al, Ga, In, and Re, significant negative (attractive) interaction energies are obtained, which is also evident from Fig. 6. Therefore, the previously discussed reduction in cycle-to-cycle variability driven by attractive dopant–vacancy interaction remains valid at lower doping concentrations, which is particularly important for dopants with a large ionic radius mismatch relative to W.

Next, we computed the tunneling probabilities at lower doping concentrations for the same dopants, excluding SOC due to its high computational cost for the large supercell and minimal impact on tunneling probability, as confirmed by our tests. To do so, we chose a 6 × 3 × 1 orthogonal supercell of WS_2_ instead of the previously chosen 3 × 3 × 1 orthogonal supercell, bringing down the doping concentration from 5.56% to 2.78%. The calculated tunneling probabilities are laid out in Table 3, alongside the values for 5.56% concentration for comparison.

**Table 3:** Tunneling probabilities in the HRS and LRS of metal-doped WS_2_ at doping concentrations of 5.56% and 2.78%

Dopant	*T* (%) (5.56% doping)		*T* (%) (2.78% doping)	
	HRS	LRS	HRS	LRS
Sr	30.38	36.39	30.94	33.78
Al	26.92	33.95	27.02	33.30
Ga	28.14	34.05	27.43	33.28
In	29.09	29.99	27.62	29.34
Ti	26.11	33.71	26.97	33.22
Zr	27.06	34.57	27.58	33.82
W (undoped)	25.21	30.97	25.37	30.68
Re	25.69	30.34	26.56	30.78

From Table 3, it can be observed that for a particular dopant and state (HRS/LRS), the *T* value does not significantly change upon reducing the doping concentration.

**Fig. 7:**
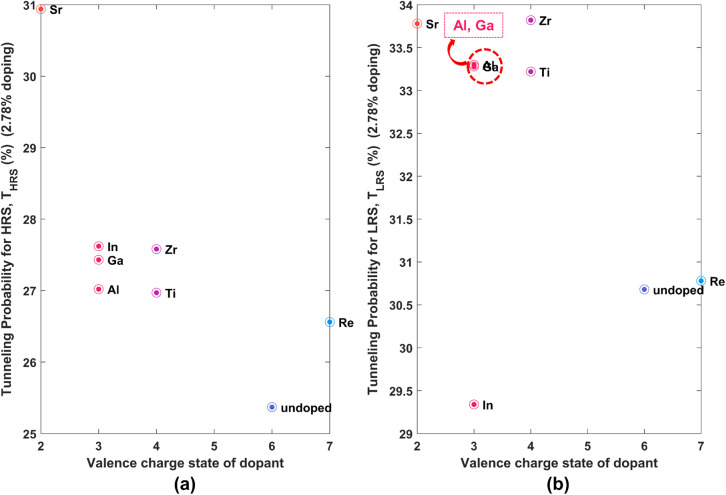
(a) and (b) Show plots of tunneling probability (*T*) against the valence charge state of dopants for HRS and LRS, respectively for lower doping concentration of 2.78%.

*T* is higher in the HRS for all 11 dopants compared to the corresponding undoped case, similar to the 5.56% case, as evident from Fig. 7(a) as well. In the case of LRS (Fig. 7(b)), the same phenomenon is observed, with the exception of the dopant In. As discussed earlier, higher values of *T* correlate to lower contact resistances, facilitating the development of more efficient, low-power devices. Thus, the implications drawn for reduced contact resistance upon doping remain valid when the doping concentration is lower than previously studied.

The results presented in this section reveal that the implications drawn for reduced cycle-to-cycle variability and reduced contact resistance upon doping remain valid when the doping concentration is lower than previously studied, which is particularly important for dopants with a large ionic radius mismatch relative to W since higher doping concentrations might not be experimentally feasible for these dopants.

### 2.7. Proposed dopant selection criteria

Based on all the results obtained so far, some dopant selection criteria are now discussed for monolayer WS_2_-based memristors to guide the choice of dopants that aid the design of improved RRAM devices. The dopant selection criteria for WS_2_ memristor based on valence charge configurations of the dopants are outlined as follows:

(1) An important outcome of doping observed from interaction energy calculations is the reduction in cycle-to-cycle variability by imposing spatial constraints on the location of vacancies through attractive interaction between metal atoms and sulphur monovacancy. In this case, stronger dopants would be preferable. Examples include Sr, Al, Ga, In, Re, and Ru. The valence charge number of W is 6, as outlined in Table S5 of the ESI.† The valence charge numbers of the dopants are as follows: Sr has a valence charge of 2; Al, Ga, and In each have a valence charge of 3; Re has a valence charge of 7; and Ru has a valence charge of 8. They have higher attractive interaction energies with the vacancy V_S_ compared to what the other dopants with closer valence charge numbers to that of W do, as outlined in Section 2.3. Therefore, the stronger dopants are preferable for realizing the desired spatial constraints and the consequent reduction in randomness and cycle-to-cycle variability.

(2) Calculations of interfacial characteristics in Section 2.4 reveal that all dopants lead to higher *T* and hence reduced contact resistance in the HRS. In the LRS, the same effect is observed, except for In. In this case, stronger p-type dopants, such as Sr, Al, and Ga are more beneficial since they lead to higher values of *T*, reduced tunneling barriers, and thereby lower contact resistances compared to the other dopants in both the HRS and the LRS.

(3) DOS calculations in Section 2.5 indicate that strong p-type and n-type dopants significantly reduce the bandgap, potentially increasing power consumption and leakage currents. Nevertheless, these dopants are more effective at reducing cycle-to-cycle variability and contact resistance, as outlined in the previous criteria. Hence, while selecting dopants, a balance must be struck between strong and weak dopants to determine the optimal choice that achieves improved device performance while maintaining tolerable power consumption.

## 3. Conclusion

Memristors are a key component in the evolution of non-volatile memory devices for neuromorphic computing. In this study, we have performed first-principles calculations of several electronic characteristics relevant to WS_2_-based memristors with and without doping. We presented several key characteristics, such as the dopant–vacancy interaction energies, tunneling barrier heights, tunneling probabilities, and density of states for the undoped and doped materials. Based on the results, we established the importance of doping in optimizing the performance of 2D WS_2_-based memristors. In the end, we outlined dopant selection guidelines based on the obtained results. These insights provide a foundation for designing next-generation WS_2_-based memristors with reduced variability and lower contact resistance.

## 4. Computational details

### 4.1 Electronic structure calculations

First-principles calculations based on density functional theory (DFT)^66,67^ were performed using the Vienna *Ab initio* Simulation Package (VASP).^68–72^ The projector-augmented wave (PAW) method^73^ and a plane-wave basis set were employed for all calculations. The Perdew–Burke–Ernzerhof (PBE) functional within generalized gradient approximation (GGA) was used for exchange-correlation energy^74^ for most calculations. However, since the PBE functional is known to underestimate the bandgap, for density of states (DOS) calculations, the Heyd–Scuseria–Ernzerhof screened hybrid functional (HSE06)^75^ was used alongside PBE. A 4 × 4 × 1 supercell of WS_2_ was used for the calculations. Thus, the concentration of sulphur monovacancy (the most relevant type of vacancy for this work) stands at 6.1467 × 10^13^ cm^−2^. This value is comparable to the experimentally measured sulphur monovacancy concentration of (3.3 ± 1.1)×10^13^ cm^−2^ in triangular monolayer WS_2_ grown *via* chemical vapour deposition (CVD).^76^ In the case of geometry optimization, atoms, and cell parameters were relaxed until residual forces were smaller than 0.01 eV Å^−1^. An energy cutoff of 400 eV was set for all computations. A 3 × 3 × 1 Monkhorst–Pack *k*-point grid was used for Brillouin zone sampling in all cases, except for the DOS, where a denser 5 × 5 × 1 Monkhorst–Pack *k*-point grid was used.

### 4.2 Formation energy

The defect (vacancy) formation energy in monolayer WS_2_ is calculated using the following equations:1
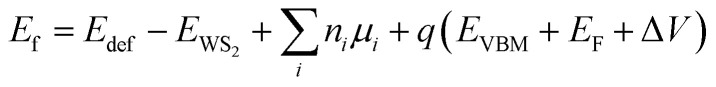

2 *μ*_W_ + 2*μ*_S_ = *μ*_WS_2__

Here, *E*_f_ is the formation energy, *E*_def_ is the energy of a defective WS_2_ supercell, and *E*_WS_2__ is the energy of a same-sized pristine WS_2_ supercell. *n*_*i*_ and *μ*_*i*_ are the count and chemical potential, respectively of atom *i* (=W or S) being doped in or removed from the system. Moreover, *q* is the defect charge state, *E*_VBM_ is the energy of valence band maximum (VBM), *E*_F_ is the Fermi level measured from VBM, and Δ*V* is the change of VBM induced by defects. The last term in eqn (2) vanishes for uncharged defects (*q* = 0), which is the kind we consider in this work. Eqn (3) imposes a restriction on the permissible values of *μ*_W_ and *μ*_S_, with their sum being fixed under the thermal equilibrium state. The sum, *μ*_WS_2__ is set equal to the energy per unit of pristine WS_2_ (=−23.66 eV). In the S-rich condition, diatomic sulphur gas is chosen as the S-reservoir, and the energy of each S atom therein is chosen as *μ*_S_ (=−3.24 eV), and using this value of *μ*_S_, *μ*_W_ (=−17.17 eV) is then calculated using eqn (2). On the other hand, the energy of each W atom of the body-centered cubic bulk W (solid) is chosen as *μ*_W_ (=−12.92 eV) in the W-rich condition, and *μ*_S_ (=−5.37 eV) is then calculated from eqn (2).

### 4.3 Interaction energy

For the doped structures, the interaction energy between vacancy and dopant atom is calculated using the following relation:^77^

3 *E*_int_ = *E*_doped,V_S__ + *E*_pristine_ − *E*_doped_ − *E*_V_S__

Here, *E*_doped,V_S__ is the total energy of a doped WS_2_ supercell with a single S vacancy, *E*_pristine_ is the total energy of a pristine 4 × 4 WS_2_ supercell, *E*_doped_ is the total energy of a doped WS_2_ supercell without S vacancy, and *E*_V_S__ is the total energy of an undoped WS_2_ supercell with a single S vacancy. The interaction energy quantifies the energetic favorability of the dopant and the vacancy being together *versus* apart. Based on this formula, the interaction energy is calculated per dopant–vacancy pair within a single supercell.

### 4.4 Migration energy barrier

To calculate migration energy barriers for Au migration, the climbing image nudged elastic band (NEB) method^78^ was used. Five intermediate images along the migration path were taken for the NEB optimization, with a force convergence criterion of 0.05 eV Å^−1^ to find the transition states and the energy barriers.

### 4.5 Tunneling barrier and probability

Tunneling barriers and probabilities were calculated for both undoped and metal-doped monolayers. At first, we computed the plane averaged effective potential across every Au/WS_2_ interface considered in this work, and then projected it in the direction of the metal–semiconductor interface using VASP and VASPKIT.^79^ This potential was then shifted with respect to the Fermi level. We label this Fermi-shifted projected potential as *V*_eff_. The barrier height (*ϕ*_TB_) was calculated from the difference between *V*_eff_ at the Au electrode and that at the interface. *ϕ*_TB_ represents the minimum energy barrier that electrons at the Fermi level must overcome for a transition to take place.^80^ The barrier width (Δ*L*) was also calculated. Compared to *ϕ*_TB_ and Δ*L*, the transmission or tunneling probability (*T*) is a better quantitative figure of merit for the strength of the contact, the electron injection rate across the interface, and the contact resistance. We computed *T* by integrating *V*_eff_ between the limits associated with the barrier width using the equation below, which comes from the WKB (Wentzel, Kramers, Brillouin) approximation:^81–84^4
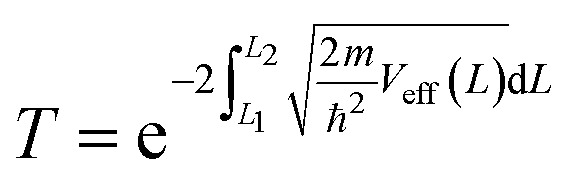
Here, *m* and *ħ* are the free electron mass and the reduced Planck's constant, respectively. To build the Au/WS_2_ interface, we took orthorhombic structures instead of the usual hexagonal ones for these calculations. A 3 × 3 × 1 orthorhombic supercell of WS_2_ was taken and sandwiched between Au(111) electrodes on both sides. This leads to a doping concentration of 5.56% (1/18) when a dopant atom is introduced in the supercell. 4 horizontal layers of Au electrode were taken on each side with 2 × 6 lateral repetition of the Au(111) orthorhombic unit cell, resulting in a lattice mismatch of ∼5%. A Monkhorst–Pack *k*-point grid of 2 × 3 × 1 was used for calculations involving the interface. van der Waals interactions were included here using Grimme's DFT-D3 method^85^ with Becke and Johnson (BJ) damping,^86^ which has the added advantage of avoiding repulsive interatomic forces at shorter distances compared to the zero-damping scheme.

### 4.6 Visualization and plotting

Crystal structures were visualized using VESTA^87^ and XCrySDen.^88^ Figures were generated using MATLAB.

## Author contributions

OH and MKA conceived the project and contributed to conceptualization, funding acquisition, project administration, resources, software, supervision, writing – review & editing. TTT contributed to conceptualization, formal analysis, investigation, methodology, visualization, writing – original draft, writing – review & editing.

## Conflicts of interest

The authors declare no competing financial or non-financial interests.

## Supplementary Material

RA-015-D5RA02473K-s001

## Data Availability

The data supporting this article have been included as part of the ESI.†
