# Supplementary material for: Doping-induced performance optimization in monolayer WS2 memristor: reduced variability and contact resistance
Source: RSC Adv. 2025 Jul 22;15(32):26052–64. doi: 10.1039/d5ra02473k (PMC12281502; doi:10.1039/d5ra02473k)
Supplement: RA-015-D5RA02473K-s001 [file RA-015-D5RA02473K-s001.pdf]

Supplementary Information (SI) for RSC Advances.
This journal is © The Royal Society of Chemistry 2025

Supporting Information

Doping-Induced Performance Optimization in Monolayer WS_2 Memristor: Reduced Variability and Contact Resistance

Tanshia Tahreen Tanisha, Orchi Hassan and Md. Kawsar Alam#*

Department of Electrical and Electronic Engineering,
Bangladesh University of Engineering and Technology, Dhaka 1205, Bangladesh

Corresponding authors' e-mails: *orchi@eee.buet.ac.bd; #kawsaralam@eee.buet.ac.bd.

A. Formation Energies of Vacancies in WS_2

We calculated the formation energies of different kinds of vacancies in monolayer WS_2 to identify which vacancy is most abundant in WS_2. We have considered W vacancy (V_W), S monovacancy (V_S), S divacancy (V_{S2}) and WS_3 vacancy (V_{WS3}) for our analyses. The V_{WS3} vacancy considered here indicates a configuration where a W atom and the three S atoms adjacent to it are absent. It is important to note that, in the case of S divacancy, the two vacancies therein are located vertically opposite to each other. Other conformations are also possible, such as cis and trans configurations.[1] However, due to the greatest energetic stability,[1] we have considered only the opposite configuration here for V_{S2}. The calculated formation energies are listed in **Table S1**.

Table S1. Formation energies, E_f of vacancies in monolayer WS_2.

	S-rich (eV)	W-rich (eV)
V_S	3.56	1.43
V_{S2}	6.94	2.68
V_W	2.26	6.51
V_{WS3}	8.35	6.22

In a practical scenario, the lower the value of the formation energy of a vacancy, the more dominant a vacancy tends to be in quantity than the other vacancies with higher formation energy. From the values of vacancy formation energies, it is apparent that V_{WS3} has the highest formation energy in the S-rich case. For the W-rich case, the value of E_f is also very high for V_{WS3}. Due to the high formation energy, V_{WS3} is ruled out for further calculations. Among the remaining three kinds of vacancies, V_S has the lowest formation energy in the W-rich case and the next-to-lowest one in the S-rich case, second to V_W. However, in the W-rich case, V_W has a much higher formation energy than V_S and V_{S2}. Lower defect formation energies translate to a greater probability of the existence of the defect and hence a greater concentration.[2] Moreover, sulphur vacancies were experimentally

found to be the most abundant ones in WS_2.[3] For these reasons, V_S is considered for our calculations.

B. Validation of Conductive-Point Mechanism in Monolayer WS_2 Memristor

Before moving on to including dopants, it is necessary to confirm whether a conductive-point or a conductive filament (or channel) is associated with the non-volatile resistive switching (NVRS) phenomenon in WS_2 memristor. At first, the following equation was used to calculate the adsorption energy of Au atoms on the WS_2 monolayer:

$$E_a = E_{WS_2+Au} - E_{WS_2} - E_{Au} \quad (S1)$$

Here, E_{WS_2+Au} is the energy of Au-adsorbed WS_2, E_{WS_2} is the energy of the corresponding WS_2 configuration without Au (with vacancy), E_{Au} is the energy of an isolated Au atom.

We found that Au atoms are adsorbed stably in the vacancies within WS_2, with significant adsorption energies of -2.3566 eV and -2.0815 eV for V_S and V_{S2} vacancies, respectively. They are of the same order as the adsorption energies of -2.543 eV and -2.398 eV obtained for MoS_2-based conductive-point memristors for V_S and V_{S2} vacancies, respectively.[4] This indicates that it is feasible to form a conductive-point in WS_2 monolayers and thus enable conductive-point switching mechanism in WS_2 memristors. Here, we considered V_S and V_{S2} vacancies only since sulphur vacancies were experimentally found to be the most dominant vacancies in WS_2, as mentioned earlier.

Next, we confirmed that the competing conductive filament switching mechanism is not feasible for WS_2-based memristors. With this end in view, the transition states and migration energy barriers have been calculated for the migration of Au atoms through V_S and V_{S2} vacancies in WS_2 monolayer. The energy landscape corresponding to the said diffusion of Au atoms is shown for V_S and V_{S2} vacancy in **Figure S1(a)** and **(b)** respectively.

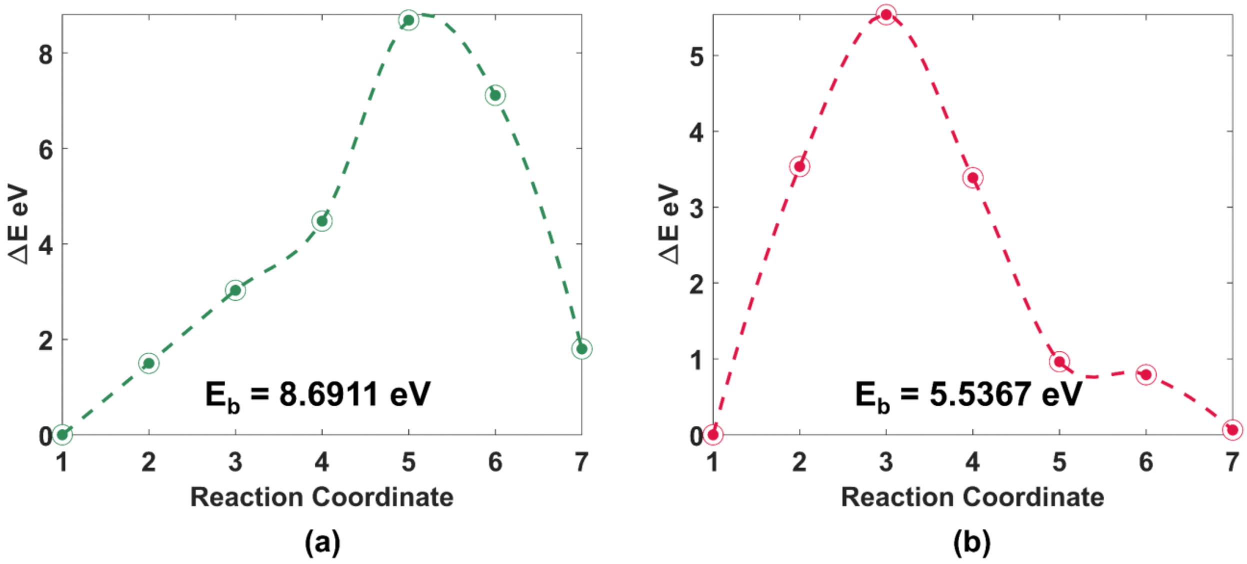

Figure S1: The energy landscape for migration of Au atoms through **(a)** V_S, **(b)** V_{S2} in WS_2 sheet.

The values of energy barriers for V_S and V_{S2} are found to be 8.6911 eV and 5.5367 eV respectively. These values are comparable to the corresponding values of 6.991 eV and 3.554 eV for MoS_2-

based conductive-point atomristors.[4] But they are quite large compared to barriers for conductive-filament based memristors, such as the 0.832 eV barrier in hBN.[5] Thus, it is evident that the formation of a full conductive filament through the migration of Au atoms through V_S and V_{S2} vacancies in the WS_2 sheet is not feasible for WS_2-based memristor having Au/WS_2/Au vertical stacking. In this case, the conductive-point mechanism is thus likely to exist, similar to the case of MoS_2.[4,6] The energies and structures corresponding to the initial, transition and final states are shown in **Table S2** for both V_S and V_{S2}.

Table S2. Energies (shifted down to the energy of the initial state) and structures corresponding to initial, transition, and final states of the diffusion pathway of migration of Au atoms through vacancies in WS_2.

Vacancy	State	Energy (eV)	Top view	Side view
	**Initial**	0.0000	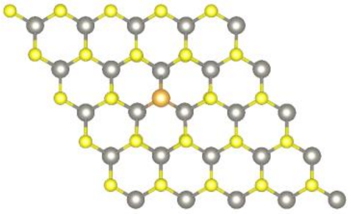	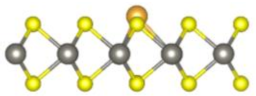
**V_S**	**Transition**	8.6911	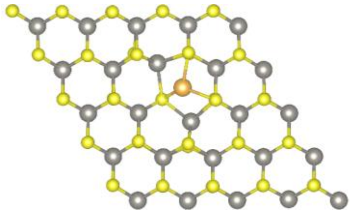	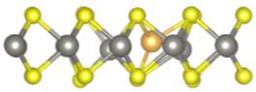
	**Final**	1.7997	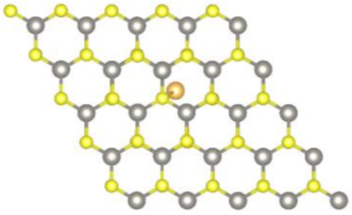	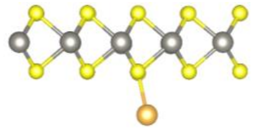
**V_{S2}**	**Initial**	0.0000	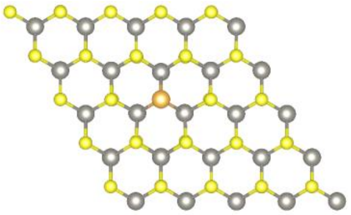	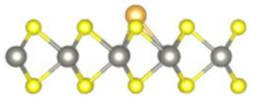
	**Transition**	5.5367	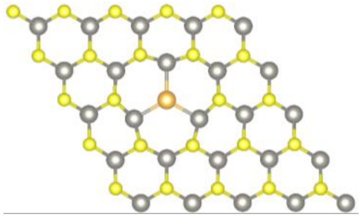	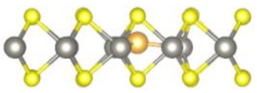

	**Final**	0.0631	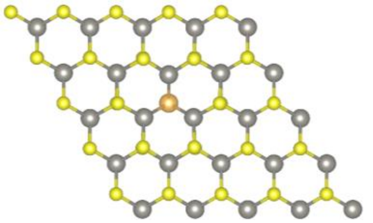	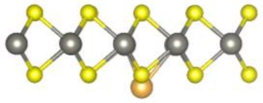

C. Ionic Radius-Based Assessment of Dopant Compatibility in Monolayer WS_2

In this section, we assessed the compatibility of Sr, Ti, Zr, and Hf with WS_2, which were not experimentally studied as dopants for monolayer WS_2 in earlier works. With this end in view, we extracted the ionic radii of these four dopants from Shannon's revised effective ionic radii (1976)[7] and compared them with that of W^{4+} in WS_2, calculating the percentage mismatches to assess their compatibility. As a benchmark, we considered the ionic radii of dopants that have been experimentally incorporated into monolayer WS_2. The extracted ionic radii for all dopants and the calculated mismatches are shown in **Table S3**.

Table S3. Ionic Radii of the Dopants and Their Differences with the Ionic Radius of W^{4+}.

Element	Ion	Ionic Radius (Å)	Mismatch with W^{4+} (%)
**Sr**	Sr^{2+}	1.18	78.8
**Al**	Al^{3+}	0.535	18.9
**Ga**	Ga^{3+}	0.62	6.1
**In**	In^{3+}	0.80	21.2
**Ti**	Ti^{4+}	0.605	8.3
**Zr**	Zr^{4+}	0.72	9.1
**Hf**	Hf^{4+}	0.71	7.6
**Nb**	Nb^{5+}	0.64	3.0
**W**	W^{4+}	0.66	0.0
**Mo**	Mo^{4+}	0.65	1.5
**Re**	Re^{4+}/Re^{7+}	0.63/0.53	4.6/19.7
**Ru**	Ru^{3+}/Ru^{4+}	0.68/0.62	3.0/6.1

In **Table S3**, the rows of experimentally unreported dopants are highlighted in light red, and the row of W in blue. It can be observed that, among the established dopants (non-highlighted rows), In^{3+} and Re^{7+} exhibit the largest mismatches, 21.2% and 19.7%, respectively, and yet both have been successfully experimentally incorporated as dopants in WS_2. In contrast, Ti^{4+} (8.3%), Zr^{4+} (9.1%), and Hf^{4+} (7.6%) show much smaller mismatches, suggesting strong potential for compatibility. In the case of Sr, the mismatch is 78.8%, which is much higher than those of the dopants with confirmed experimental incorporation. However, Sr has been used as a substitutional dopant for two-dimensional TMDC $MoSe_2$ nanosheets,[8] where the Mo^{4+} cation in the host has a radius very close to that of W^{4+}, which suggests the potential compatibility of Sr with WS_2 for

substitutional doping. Therefore, all of the four dopants, Sr, Ti, Zr, or Hf might be compatible with monolayer WS_2 doping.

It is important to note here, the oxidation number of W in WS_2 is 4+[13] and the co-ordination number is 6 since it is surrounded by six nearest neighbor sulphur anions. These two pieces of information were used for extracting the ionic radius of W^{4+} based on Shannon's revised effective ionic radii. For the dopant metal ions, the co-ordination number is 6, just like W^{4+}. However, the most stable and commonly observed oxidation number(s) of each dopant ion is (are) chosen. In case of Re, 4+ and 7+ oxidation states are common,[14] and thus they are both tabulated and compared. Similarly, for Ru, both 3+ and 4+ oxidation states are common[15] and thus taken into consideration. 2+ is also common for Ru[15] but data was not available for this state.

It is relevant to note here, significant progress has been made in synthesizing doped WS_2 monolayers through techniques like one-step CVD using IIIA elements (e.g., B, Ga, In), which allow control over both photoluminescence and carrier polarity.[12] Liquid-phase precursor mixing has enabled Nb doping with high p-type doping concentrations.[10] Different CVD-based methods, such as two-step CVD, in-situ CVD, etc. have been developed to achieve dopant incorporation of elements like Mo, Yb, and Re.[9,11,16] Thus, it might be possible to incorporate the other dopants we propose here i.e. Sr, Ti, Zr, and Hf through novel synthesis methods. Despite these advances, key challenges in synthesis persist, including achieving uniform dopant distribution, maintaining control over doping level and type, and avoiding growth-induced strain and lattice distortions from dopant-host size mismatch.[10,12,16] Our ionic radius-based assessment can serve as a practical tool for predicting dopant compatibility and guiding future experimental efforts in WS_2 monolayer doping. The calculated lower difference in ionic radii could be a reason why Mo and Nb-doped WS_2 were realized at higher doping concentrations (1 to 100%: full range, >10% respectively) experimentally.[9,10] On the other hand, since In and Re have large mismatches with W, In and Re-doped WS_2 were realized at comparatively lower doping concentrations of ~0.258% and ~1%, respectively in experimental works.[11,12] This coherence indicates the reliability of our analytical approach.

D. Site Selection for Metal Dopants in Monolayer WS_2

In order to identify the most energetically favorable site for positioning the metal dopant atom in the WS_2 supercell, we calculated the energies of 16 different doped WS_2 configurations where the dopant is placed in all the 16 different possible sites. We repeated this for each metal dopant. The 16 different sites in a WS_2 supercell with single S-vacancy are illustrated in **Figure S2**. The calculated energies are listed in **Table S4**.

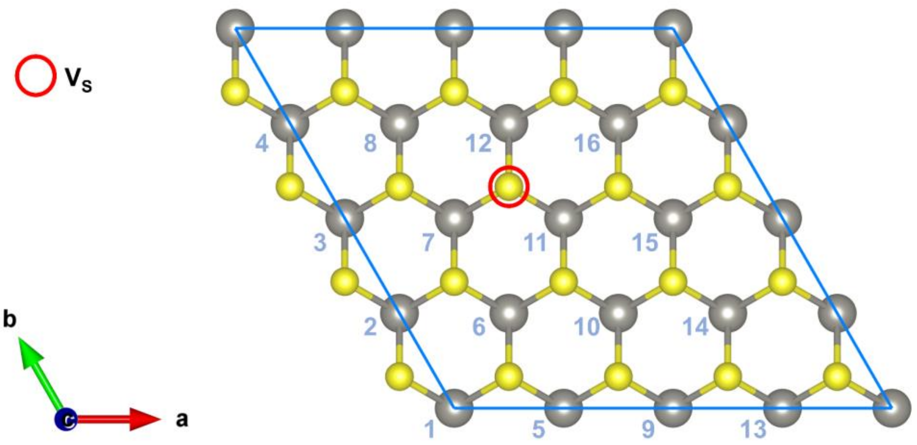

Figure S2: Top view of a 4 × 4 × 1 WS_2 supercell showing all 16 possible sites for dopant incorporation, labeled 1-16.

Table S4. Energies of Doped WS_2 with the Metal Dopant Atom Placed in 16 Different Sites.

Metals	Sites															
	1	2	3	4	5	6	7	8	9	10	11	12	13	14	15	16
	Energies (eV)															
Sr	-356.45	-357.40	-357.41	-356.44	-356.44	-356.32	-358.43	-356.27	-357.38	-357.37	-358.44	-358.45	-357.42	-356.61	-357.39	-356.30
Al	-359.35	-359.59	-359.55	-359.34	-359.31	-359.29	-360.64	-359.28	-359.55	-359.59	-360.65	-360.64	-359.59	-359.32	-359.59	-359.28
Ga	-357.00	-357.31	-357.30	-356.97	-356.96	-356.93	-358.30	-356.91	-357.33	-357.32	-358.31	-358.30	-357.32	-356.96	-357.32	-356.91
In	-356.12	-356.45	-356.45	-356.12	-356.11	-355.98	-357.42	-356.00	-356.43	-356.40	-357.44	-357.43	-356.44	-356.13	-356.45	-355.94
Ti	-366.80	-366.85	-366.81	-366.71	-366.72	-366.72	-366.96	-366.67	-366.85	-366.86	-366.93	-366.96	-366.85	-366.72	-366.81	-366.70
Zr	-367.66	-367.94	-367.81	-367.65	-367.65	-367.60	-367.79	-367.62	-367.85	-367.85	-367.77	-367.79	-367.86	-367.71	-367.85	-367.59
Hf	-369.17	-369.41	-369.33	-369.13	-369.18	-369.14	-369.20	-369.17	-369.34	-369.41	-369.21	-369.22	-369.34	-369.20	-369.34	-369.14

Metals	Sites															
	1	2	3	4	5	6	7	8	9	10	11	12	13	14	15	16
**Nb**	-369.45	-369.49	-369.59	-369.45	-369.44	-369.44	-369.44	-369.45	-369.45	-369.45	-369.50	-369.50	-369.50	-369.46	-369.50	-369.38
**W (undoped)**	-371.68	-371.68	-371.68	-371.68	-371.68	-371.68	-371.68	-371.68	-371.68	-371.68	-371.68	-371.68	-371.68	-371.68	-371.68	-371.68
**Mo**	-369.82	-369.96	-369.85	-369.82	-369.82	-369.82	-369.84	-369.77	-369.81	-369.86	-369.83	-369.84	-369.86	-369.79	-369.86	-369.82
**Re**	-369.56	-369.64	-369.62	-369.52	-369.52	-369.55	-370.05	-369.55	-369.58	-369.62	-370.10	-370.11	-369.61	-369.45	-369.58	-369.55
**Ru**	-365.02	-365.24	-365.23	-364.96	-365.00	-365.23	-366.67	-365.27	-365.23	-365.22	-366.67	-366.67	-365.32	-365.23	-365.23	-365.28

The sites corresponding to lowest energy are highlighted in blue in **Table S4**. Here, sites 11 and 12 are equivalent. The configurations with the dopant atom placed in the lowest energy site are considered for further calculations.

E. Values of Dopant-Vacancy Interaction Energy in the Doped and Undoped Structures

Table S5. Values of dopant-vacancy interaction energy (with SOC) in doped and undoped WS_2.

Dopant	Outermost electron configuration	Number of valence electrons	Dopant type	E_{int} (with SOC) (eV)
**Sr**	$5s^2$	2	p-type	-1.7104
**Al**	$3s^23p^1$	3	p-type	-1.1822
**Ga**	$4s^24p^1$	3	p-type	-1.2435
**In**	$5s^25p^1$	3	p-type	-1.1510
**Ti**	$3d^24s^2$	4	p-type	-0.1051
**Zr**	$4d^25s^2$	4	p-type	-0.0695
**Hf**	$5d^26s^2$	4	p-type	-0.0415
**Nb**	$4d^45s^1$	5	p-type	-0.0052
**W (undoped)**	$5d^46s^2$	6	W-like	0.0000
**Mo**	$4d^55s^1$	6	W-like	-0.0161
**Re**	$5d^56s^2$	7	n-type	-0.9094
**Ru**	$4d^75s^1$	8	n-type	-1.9192

F. Structures Used to Calculate the Tunneling Barrier and Probability (T) of the Doped Structures in the HRS and the LRS

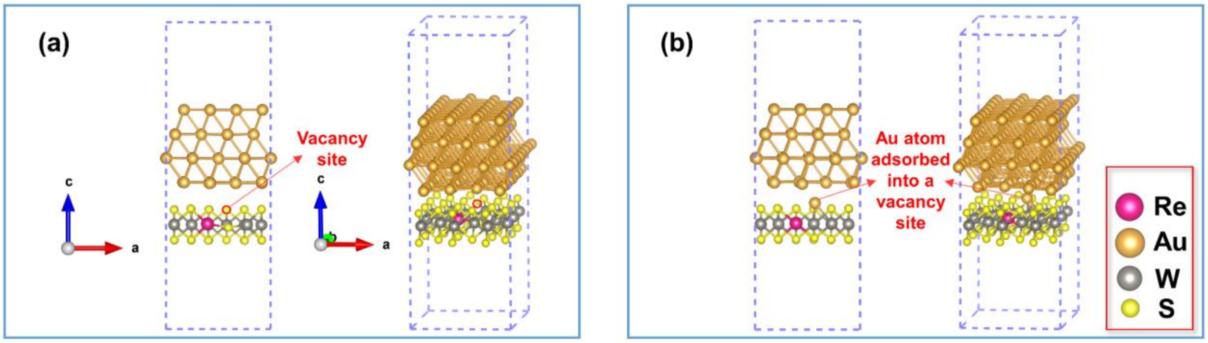

Figure S3: The structures used to calculate the tunneling barrier and probability for metal-doped WS_2 in HRS and LRS are shown in **(a)** and **(b),** respectively. These configurations are shown for the representative case of Re dopant.

G. Plots of Tunneling Probability (T) for Different Dopants

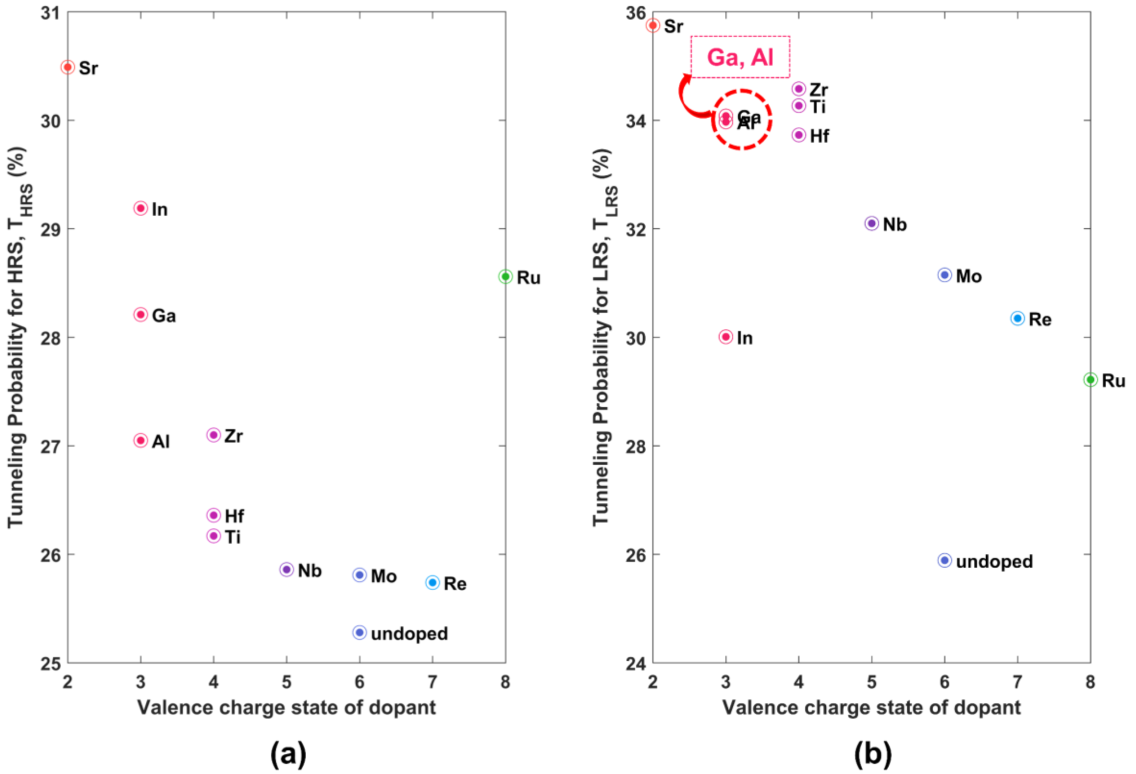

Figure S4: (a) and **(b)** show plots of tunneling probability (T) with SOC against the valence charge state of dopants for HRS and LRS, respectively.

H. Plots of DOS Computed with GGA and GGA+SOC

Table S6. DOS (with E-E_F along the x-axis) corresponding to the HRS of metal-doped WS_2, with and without SOC. Extra defect states induced by the dopants are highlighted with circles, and the number of such states is listed as n_{ex}.

Dopant	Without SOC	With SOC	n_{ex}
(a) W (un-doped)	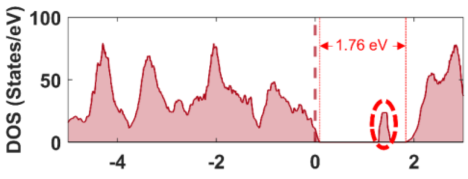	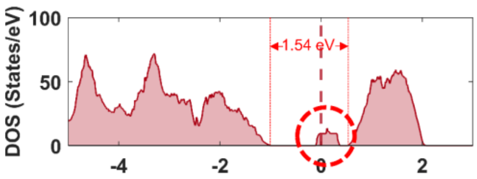	1
(b) Mo	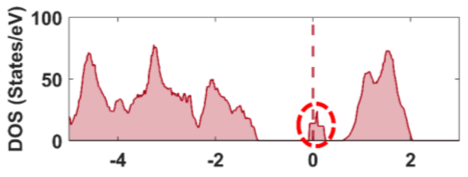	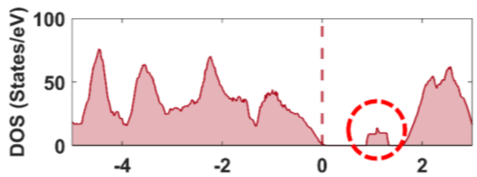	1
(c) Re	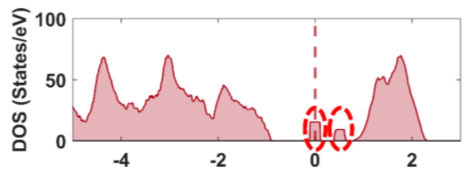	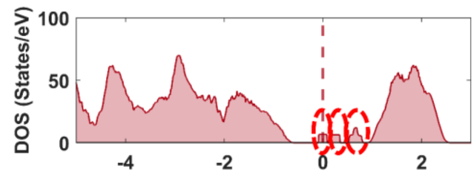	2,3
(d) Ru	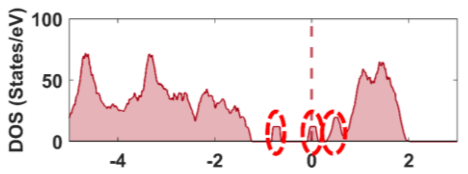	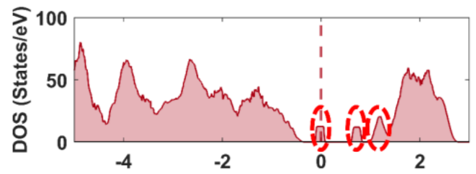	3
(e) Nb	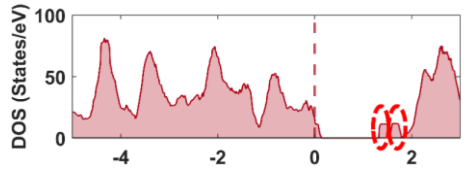	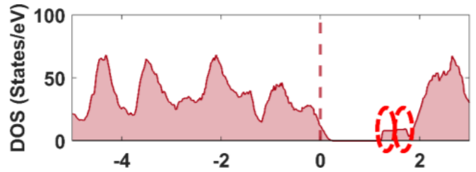	2
(f) Ti	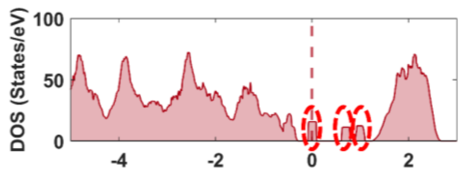	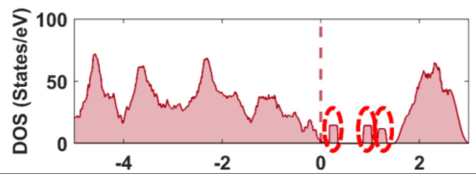	3
(g) Zr	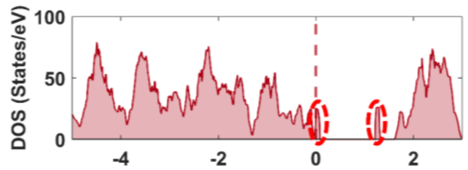	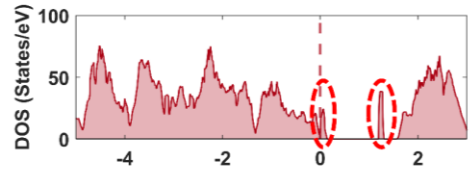	2
(h) Hf	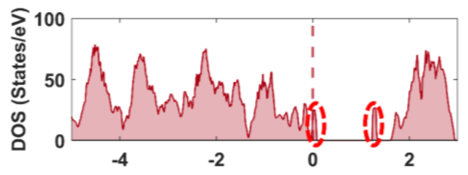	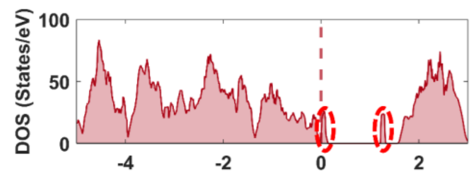	2

Dopant	Without SOC	With SOC	n_{ex}
(i) In	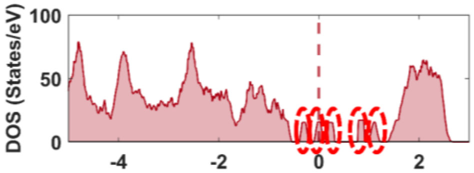	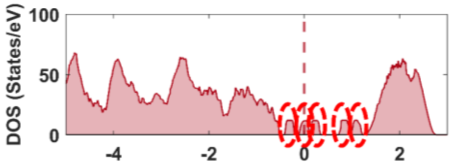	5
(j) Ga	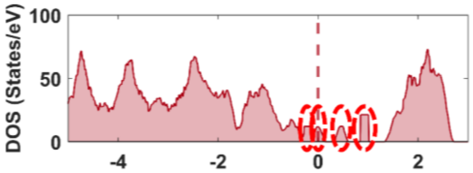	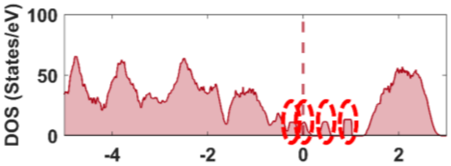	4
(k) Al	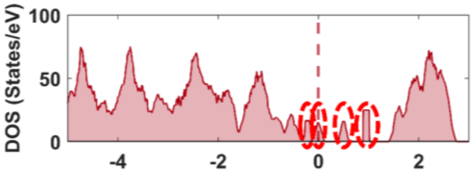	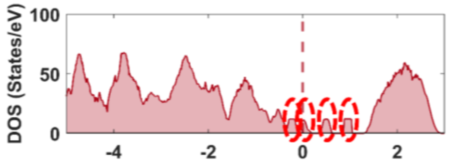	4
(l) Sr	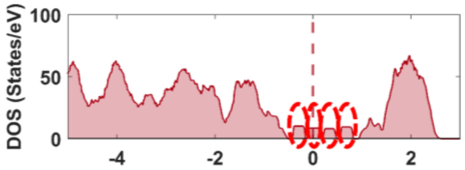	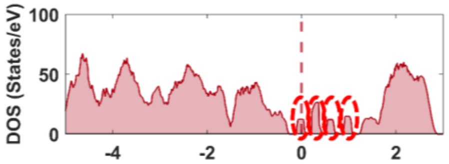	4

Table S7. DOS (with $E-E_F$ along the x-axis) corresponding to the LRS of metal-doped WS_2, with and without SOC. Extra defect states induced by the dopants are highlighted with circles, and the number of such states is listed as n_{ex}.

Dopant	Without SOC	With SOC	n_{ex}
(a) W (un-doped)	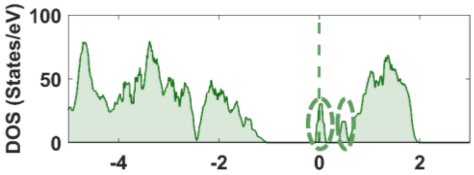	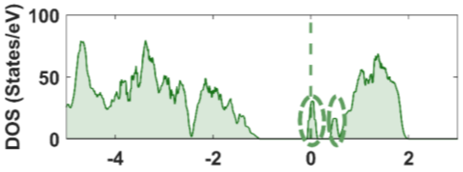	2
(b) Mo	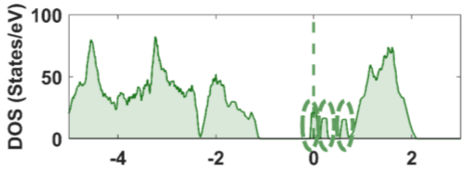	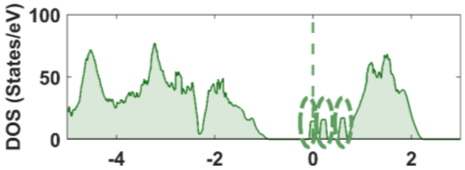	3
(c) Re	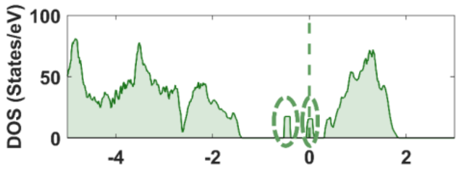	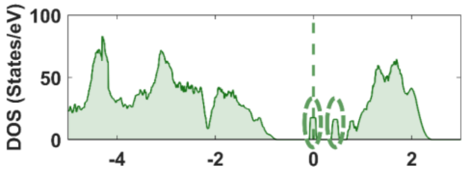	2

Dopant	Without SOC	With SOC	n_{ex}
(d) Ru	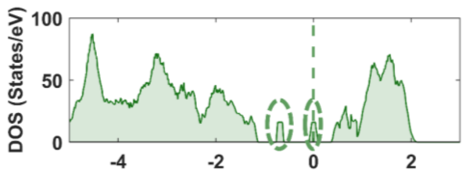	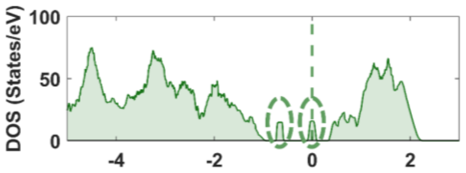	2
(e) Nb	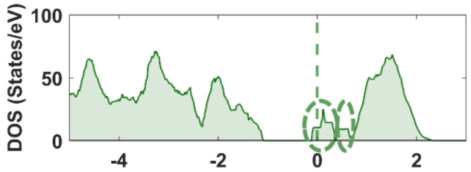	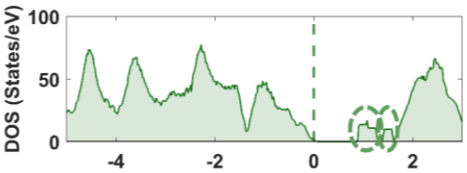	2
(f) Ti	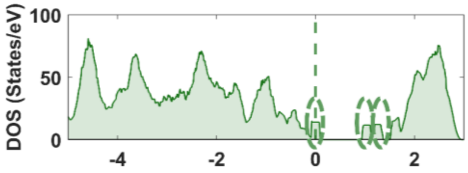	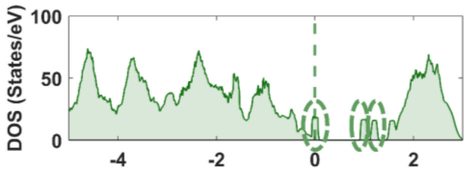	3
(g) Zr	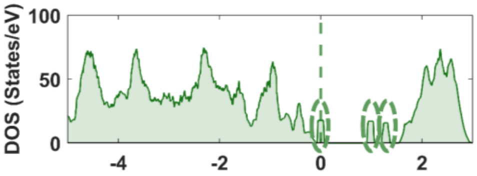	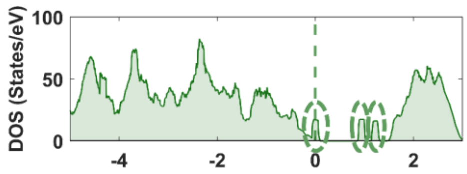	3
(h) Hf	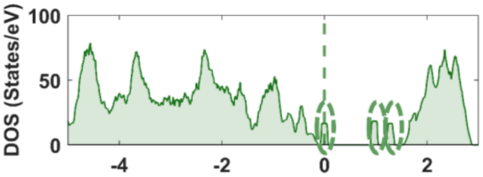	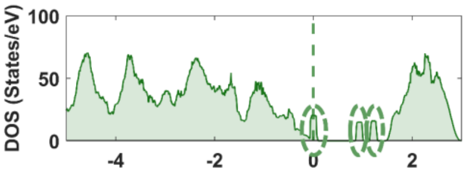	3
(i) In	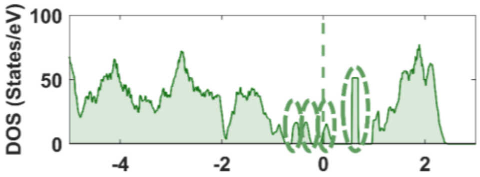	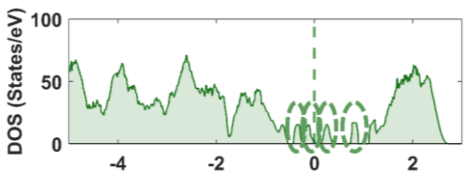	4
(j) Ga	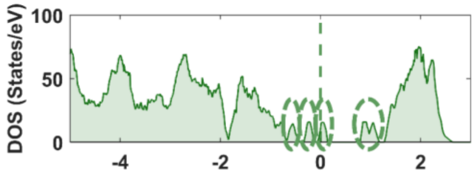	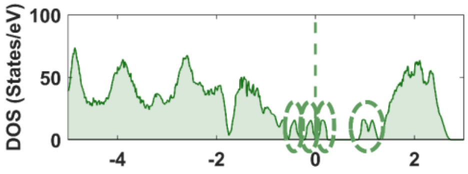	4
(k) Al	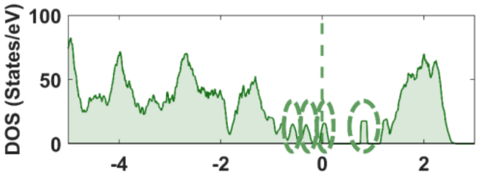	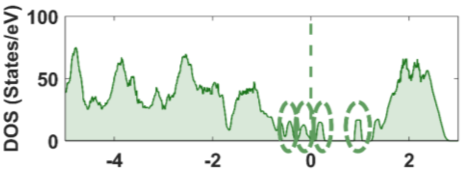	4
(l) Sr	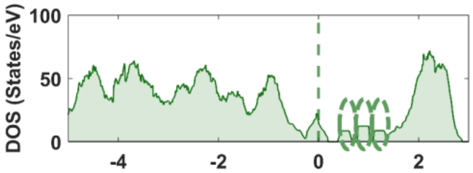	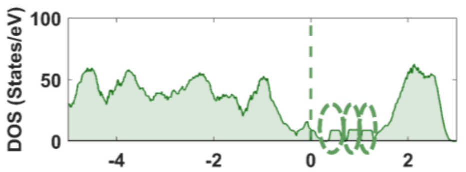	3

I. Plots of DOS Computed with HSE06 Functional

The computed DOS for the HRS and LRS with HSE06 functional (without SOC) are shown in **Figure S5** and **Figure S6,** respectively. They are similar to the ones computed with PBE, with a single exception. In the LRS, Mo shows three and two states in case of PBE and HSE respectively. Other than this, all the other observations obtained for the PBE-computed DOS are applicable for the HSE06-computed DOS as well.

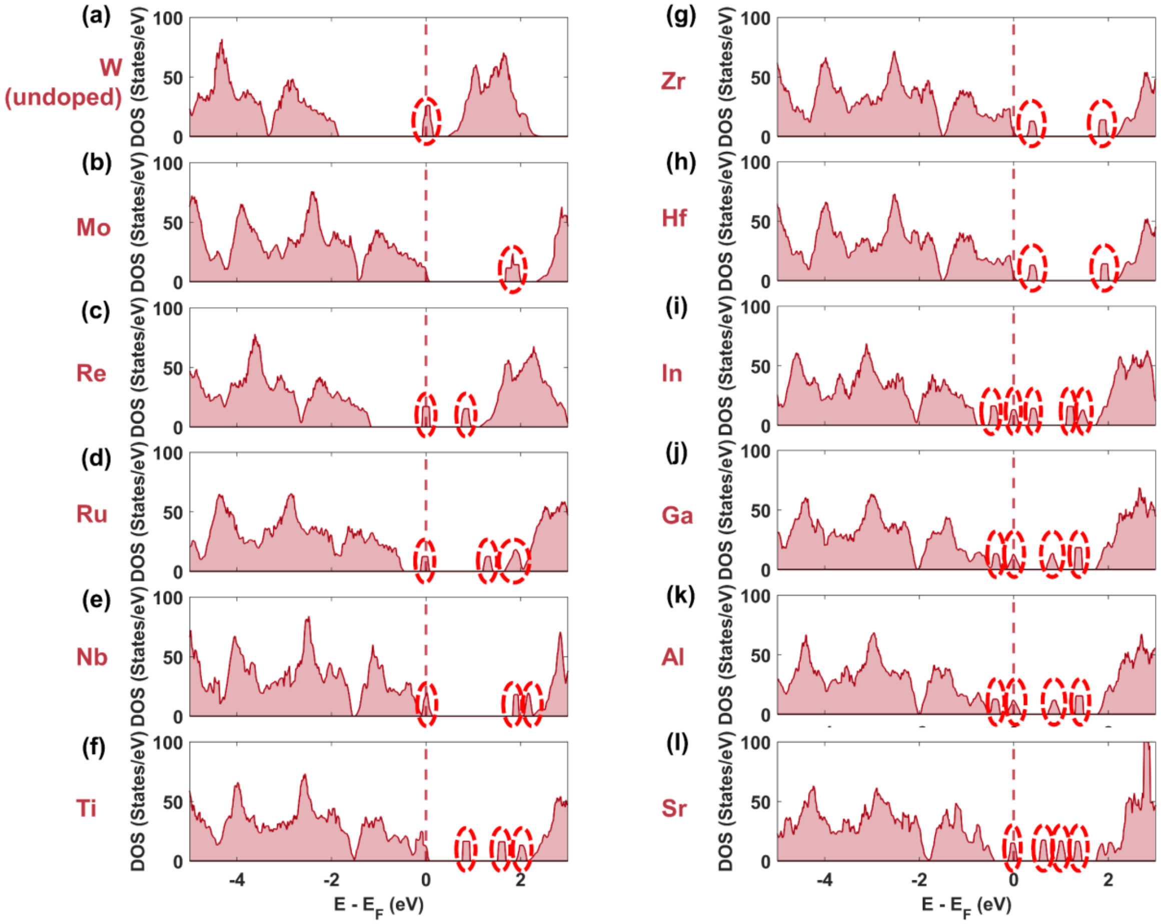

Figure S5: Density of states (DOS) using HSE06 functional for WS_2 with a single V_S doped with **(a)** W i.e. without dopant, **(b)** Mo, **(c)** Re, **(d)** Ru, **(e)** Nb, **(f)** Ti, **(g)** Zr, **(h)** Hf, **(i)** In, **(j)** Ga, **(k)** Al, and **(l)** Sr. These DOS plots correspond to the HRS of WS_2 memristor when it is doped with the said metal atoms. The defect states arising in the DOS due to dopants are encircled in red in **Figure S5 (a)-(l)**.

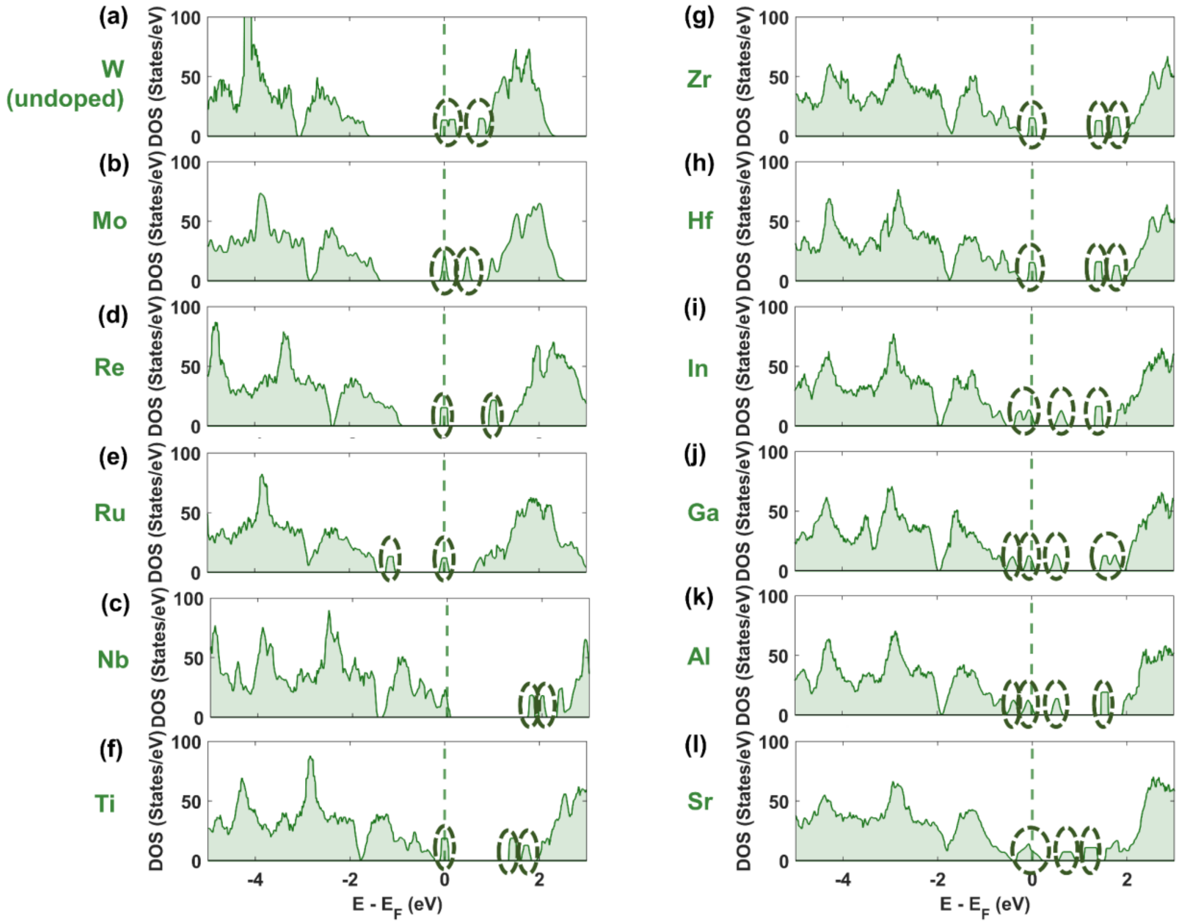

Figure S6: Density of states (DOS) using HSE06 functional for WS_2 with an Au atom adsorbed into a single V_S, when WS_2 is doped with **(a)** W i.e. without dopant, **(b)** Mo, **(c)** Re, **(d)** Ru, **(e)** Nb, **(f)** Ti, **(g)** Zr, **(h)** Hf, **(i)** In, **(j)** Ga, **(k)** Al, and **(l)** Sr. These DOS plots correspond to the LRS of WS_2 memristor when it is doped with the said metal atoms. The defect states arising in the DOS due to dopants are encircled in green in **Figure S6 (a)-(l)**.

J. References

(1) Yan, X.; Zhao, Q.; Chen, A. P.; Zhao, J.; Zhou, Z.; Wang, J.; Wang, H.; Zhang, L.; Li, X.; Xiao, Z.; Wang, K.; Qin, C.; Wang, G.; Pei, Y.; Li, H.; Ren, D.; Chen, J.; Liu, Q. Vacancy-Induced Synaptic Behavior in 2D WS2 Nanosheet–Based Memristor for Low-Power Neuromorphic Computing. *Small* **2019**, *15* (24), 1901423. https://doi.org/10.1002/smll.201901423.

(2) Bertoldo, F.; Ali, S.; Manti, S.; Thygesen, K. S. Quantum Point Defects in 2D Materials - the QPOD Database. *npj Comput. Mater.* **2022**, *8* (1), 56. https://doi.org/10.1038/s41524-022-00730-w.

(3) Jeong, T. Y.; Kim, H.; Choi, S.-J.; Watanabe, K.; Taniguchi, T.; Yee, K. J.; Kim, Y.-S.;

Jung, S. Spectroscopic Studies of Atomic Defects and Bandgap Renormalization in Semiconducting Monolayer Transition Metal Dichalcogenides. *Nat. Commun.* **2019**, *10* (1), 3825. https://doi.org/10.1038/s41467-019-11751-3.

(4) Li, X.-D.; Wang, B.-Q.; Chen, N.-K.; Li, X.-B. Resistive Switching Mechanism of MoS2 Based Atomristor. *Nanotechnology* **2023**. https://doi.org/10.1088/1361-6528/acb69d.

(5) Li, X.-D.; Chen, N.-K.; Wang, B.-Q.; Li, X.-B. Conductive Mechanism in Memristor at the Thinnest Limit: The Case Based on Monolayer Boron Nitride. *Appl. Phys. Lett.* **2022**, *121* (7). https://doi.org/10.1063/5.0098120.

(6) Ge, R.; Wu, X.; Liang, L.; Hus, S. M.; Gu, Y.; Okogbue, E.; Chou, H.; Shi, J.; Zhang, Y.; Banerjee, S. K.; Jung, Y.; Lee, J. C.; Akinwande, D. A Library of Atomically Thin 2D Materials Featuring the Conductive-Point Resistive Switching Phenomenon. *Adv. Mater.* **2021**, *33* (7). https://doi.org/10.1002/adma.202007792.

(7) Shannon, R. D. Revised Effective Ionic Radii and Systematic Studies of Interatomic Distances in Halides and Chalcogenides. *Acta Crystallogr. Sect. A* **1976**, *32* (5), 751–767. https://doi.org/10.1107/S0567739476001551.

(8) Sakthivel, M.; Sukanya, R.; Chen, S.-M.; Dinesh, B. Synthesis of Two-Dimensional Sr-Doped MoSe 2 Nanosheets and Their Application for Efficient Electrochemical Reduction of Metronidazole. *J. Phys. Chem. C* **2018**, *122* (23), 12474–12484. https://doi.org/10.1021/acs.jpcc.8b02188.

(9) Bogaert, K.; Liu, S.; Liu, T.; Guo, N.; Zhang, C.; Gradečak, S.; Garaj, S. Two-Dimensional MoxW1−xS2 Graded Alloys: Growth and Optical Properties. *Sci. Rep.* **2018**, *8* (1), 12889. https://doi.org/10.1038/s41598-018-31220-z.

(10) Qin, Z.; Loh, L.; Wang, J.; Xu, X.; Zhang, Q.; Haas, B.; Alvarez, C.; Okuno, H.; Yong, J. Z.; Schultz, T.; Koch, N.; Dan, J.; Pennycook, S. J.; Zeng, D.; Bosman, M.; Eda, G. Growth of Nb-Doped Monolayer WS 2 by Liquid-Phase Precursor Mixing. *ACS Nano* **2019**, *13* (9), 10768–10775. https://doi.org/10.1021/acsnano.9b05574.

(11) Loh, L.; Chen, Y.; Wang, J.; Yin, X.; Tang, C. S.; Zhang, Q.; Watanabe, K.; Taniguchi, T.; Wee, A. T.; Bosman, M.; Quek, S. Y.; Eda, G. Impurity-Induced Emission in Re-Doped WS 2 Monolayers. *Nano Lett.* **2021**, *21* (12), 5293–5300. https://doi.org/10.1021/acs.nanolett.1c01439.

(12) Chen, Y.; Liu, H.; Yu, G.; Ma, C.; Xu, Z.; Zhang, J.; Zhang, C.; Chen, M.; Li, D.; Zheng,

W.; Luo, Z.; Yang, X.; Li, K.; Yao, C.; Zhang, D.; Xu, B.; Yi, J.; Yi, C.; Li, B.; Zhang, H.; Zhang, Z.; Zhu, X.; Li, S.; Chen, S.; Jiang, Y.; Pan, A. Defect Engineering of 2D Semiconductors for Dual Control of Emission and Carrier Polarity. *Adv. Mater.* **2024**, *36* (14). https://doi.org/10.1002/adma.202312425.

(13) Kumar, P.; Singh, M.; Reddy, G. B. Core–Shell WO 3 –WS 2 Nanostructured Thin Films via Plasma Assisted Sublimation and Sulfurization. *ACS Appl. Nano Mater.* **2019**, *2* (3), 1691–1703. https://doi.org/10.1021/acsanm.9b00136.

(14) Housecroft, C. *Inorganic Chemistry*, 5th ed.; Pearson, 2018.

(15) Boglaienko, D.; Hall, G. B.; D'Annunzio, N. L.; Levitskaia, T. G. Ruthenium Speciation and Distribution in the Environment: A Review. *Sci. Total Environ.* **2024**, *951*, 175629. https://doi.org/10.1016/j.scitotenv.2024.175629.

(16) Liu, S.; Zhao, Y.; Cao, S.; Chen, S.; Wang, C.; Shi, X.; Zhao, H. High Photoresponse Detectors Based on Yb-Doped Monolayer WS2 Nanosheets. *Appl. Surf. Sci.* **2024**, *652*, 159287. https://doi.org/10.1016/j.apsusc.2024.159287.
